# Mannosyltransferase (GPI-14) overexpression protects promastigote and amastigote forms of *Leishmania braziliensis* against trivalent antimony

**DOI:** 10.1186/s13071-019-3305-2

**Published:** 2019-01-25

**Authors:** Christiana Vargas Ribeiro, Bruna Fonte Boa Rocha, Douglas de Souza Moreira, Vanessa Peruhype-Magalhães, Silvane Maria Fonseca Murta

**Affiliations:** Instituto René Rachou IRR, Fundação Oswaldo Cruz - FIOCRUZ/Minas, Avenida Augusto de Lima, Belo Horizonte, MG 1715 Brazil

**Keywords:** *Leishmania braziliensis*, Glycosylphosphatidylinositol, Mannosyltransferase, Antimony resistance, Concanavalin-A

## Abstract

**Background:**

Glycosylphosphatidylinositol is a surface molecule important for host-parasite interactions. Mannosyltransferase (GPI-14) is an essential enzyme for adding mannose on the glycosylphosphatidyl group. This study attempted to overexpress the *GPI-14* gene in *Leishmania braziliensis* to investigate its role in the antimony-resistance phenotype of this parasite.

**Results:**

*GPI-14* mRNA levels determined by quantitative real-time PCR (qRT-PCR) showed an increased expression in clones transfected with GPI-14 compared to its respective wild-type line. In order to investigate the expression profile of the surface carbohydrates of these clones, the intensity of the fluorescence emitted by the parasites after concanavalin-A (a lectin that binds to the terminal regions of α-D-mannosyl and α-D-glucosyl residues) treatment was analyzed. The results showed that the clones transfected with GPI-14 express 2.8-fold more mannose and glucose residues than those of the wild-type parental line, indicating effective GPI-14 overexpression. Antimony susceptibility tests using promastigotes showed that clones overexpressing the GPI-14 enzyme are 2.4- and 10.5-fold more resistant to potassium antimonyl tartrate (Sb^III^) than the parental non-transfected line. Infection analysis using THP-1 macrophages showed that amastigotes from both GPI-14 overexpressing clones were 3-fold more resistant to Sb^III^ than the wild-type line.

**Conclusions:**

Our results suggest the involvement of the GPI-14 enzyme in the Sb^III^-resistance phenotype of *L. braziliensis*.

## Background

Leishmaniasis is an important neglected tropical disease caused by at least 21 different species of protozoan parasites belonging to the genus *Leishmania*. The three main clinical forms of this disease are visceral (VL), cutaneous (CL) and mucocutaneous (MCL) [1, 2]. *Leishmania* (*Viannia*) *braziliensis*, which is broadly distributed in the Americas, is the etiological agent of both CL and MCL [3, 4]. It is estimated that 700,000 to one million new cases and 20,000 to 30,000 deaths occur annually due to leishmaniasis [2]. CL is more widely distributed, with ten countries presenting the highest number of cases: Afghanistan, Algeria, Colombia, Brazil, Iran, Syria, Ethiopia, North Sudan, Costa Rica and Peru. Together, these countries account for 70 to 75% of CL cases worldwide [5].

There is no vaccine available for humans; hence, chemotherapy is the main form of leishmaniasis control [6]. Pentavalent antimonials (Sb^V^) have been the drugs of first choice for the treatment in many countries for more than 70 years [7]. In addition to their toxicity, pentavalent antimonials require long treatment schedules and present high rates of treatment failure (above 65%) in some places, such as the State of Bihar in India [8, 9]. The mode of antimony action is still not completely understood. It is accepted that Sb^V^ is a prodrug that is reduced to the trivalent (Sb^III^) form with a leishmanicidal effect against amastigote and promastigote forms of the parasite [10]. Some studies have indicated that Sb^V^ inhibits glycolysis and fatty acid oxidation [11]. Different antimonial-resistance mechanisms have been proposed, such as increased efflux/sequestration of active molecules, decreased drug reduction/activation, lower uptake, gene amplification and higher activity of repair mechanisms for damage caused by the drug [7].

Lipophosphoglycan (LPG) and glycoinositolphospholipids (GIPLs) are glycosylphosphatidylinositol (GPI) anchored molecules that form a protective surface coat and mediate essential host-parasite interactions [12]. Mannosyltransferase (GPI-14) is an essential enzyme for adding mannose on glycosylphosphatidyl. Mannose plays a key role in maintaining the energy and redox balance of *Leishmania* as well as increasing its virulence in the vector and macrophages [13]. GPI-14 is functionally different from that of the mammalian pathway. Structural variations in the side chain and lipid moiety between *Leishmania* and humans make GPI-14 a rational drug target [14]. In addition, this enzyme can be a good target for antiparasitic chemotherapy due to its role in the biosynthesis of LPG and GIPLs, which are important molecules involved in the parasite’s infection cycle. To the best of our knowledge, the role of GPI-14 on drug resistance mechanisms is not yet known. Thus, this study attempts to overexpress the *GPI-14* gene in *L. braziliensis* to investigate the contribution of this enzyme in the antimony-resistance phenotype of this parasite.

## Methods

Promastigotes of *Leishmania* (*Viannia*) *braziliensis* (MHOM/BR/75/M2904) were grown at 26 °C in M199 medium supplemented as previously described [15]. All analyses were performed with parasites in the exponential growth phase.

In order to generate overexpression, a 1299 bp fragment corresponding to *GPI-14* encoding region (open reading frame-ORF) (TriTrypDB accession number LbrM.30.1970) was amplified with *Pfx* DNA polymerase (Invitrogen, Carlsbad, USA) from *L. braziliensis* genomic DNA using the forward primer 5'-TGG ATC C**CC ACC** ATG AGC AAG GCA ACG TGG C-3' and the reverse primer 5'-TTG GAT CCC TAA ACC TCC TTG CGC GTC-3'. Bold letters indicate the Kozak sequence and the underlined sequences correspond to the *BamH*I restriction site. The next steps were performed as previously reported [16]. Briefly, the GPI-14 1299 bp fragment was cloned into a pGEM-T Easy® vector (Promega, Madison, WI, USA) and confirmed by sequencing. Subsequently, pGEM-GPI-14 was digested with *BamH*I enzyme and introduced into dephosphorylated pIR1BSD expression vector (kindly provided by Dr Stephen Beverley, Washington University, USA). Next, the constructs pIR1BSD (empty vector) and pIR1BSD-GPI-14 were linearized upon *Swa*I digestion, electroporated into wild-type *L. braziliensis*, and the colonies were obtained on semisolid M199 medium containing 10 μg/ml blasticidin (BSD).

Quantitative real time PCR (qRT-PCR) analysis was performed to investigate the levels of *GPI-14* mRNA in *Leishmania* clones, as described previously [17]. The amount of *GPI-14* cDNA in each sample was normalized to that of the *DNA polymerase* gene.

In order to investigate the expression profile of the surface carbohydrates of these GPI-14-transfected clones, the mean fluorescence intensity was analyzed by flow cytometry of the parasites incubated with the concanavalin-A (Con-A), a lectin that binds to the terminal regions of α-D-mannosyl and α-D-glucosyl residues. Briefly, promastigotes of *L. braziliensis* samples in the stationary growth phase (2 × 10^6^ parasites/ml) were washed with PBS and incubated with Con-A lectin conjugated to fluorescein isocyanate (FITC) (Vector Laboratories, Burlingame, CA, USA) at a final concentration of 10 μg/ml for 30 min at 37 °C in a 5% CO_2_ incubator. Next, the parasites were acquired by flow cytometer (Fortessa LSR, Becton Dickinson BD, Franklin Lakes, USA) and the data were analyzed using FlowJo v.10 software. The geometric mean fluorescence intensity (gMFI) and the Con-A-labeled-*Leishmania* percentage (%) for each *L. braziliensis* sample were determined.

Promastigotes of wild-type *L. braziliensis* and GPI-14-overexpression cell lines were incubated in M199 medium at 2 × 10^6^ cells/ml in 24-well plates in the absence or presence of increasing concentrations (1.2–74.9 μM) of potassium antimonyl tartrate (Sb^III^) (Sigma-Aldrich, St. Louis, MO, USA) for 48 h. The effective concentration required to decrease growth by 50% (EC_50_) was determined using a model Z1 Coulter Counter (Beckman Coulter, Fullerton, CA, USA).

Amastigotes of GPI-14-overexpressing *L. braziliensis* clones were also subjected to susceptibility assays with Sb^III^ to analyze whether the antimony-resistant phenotype persists in parasite’s intracellular form. Briefly, human macrophages differentiated from THP-1 cells (ATCC TIB 202) were seeded (4 × 10^5^ cells/well) on a 13 mm coverslip placed inside 24-well plates for 72 h at 37 °C, in a 5% CO_2_ atmosphere for macrophage adherence. Then, the adhered macrophages were exposed to stationary phase promastigote *L. braziliensis* samples (4 × 10^6^ parasites/well) (10:1 parasites/macrophage). After 5 h of infection, the free parasites were removed and RPMI medium was added in the absence or presence of Sb^III^ at a concentration ranging from 12.5 to 200 μM. After 72 h of incubation, adhered macrophages were stained by the panoptic method. The infected cells and the number of intracellular amastigotes were determined using ImageJ software (v.1.50i, Wayne Rasband National Institute of Health). EC_50_ values were obtained from three independent measurements in triplicate using the linear interpolation method [18].

### Statistical analysis

Data were analyzed using Student’s t-test, performed using the software GraphPad Prism v.5.0. A *P-*value less than 0.05 was considered statistically significant.

## Results

**Fig. 1:**
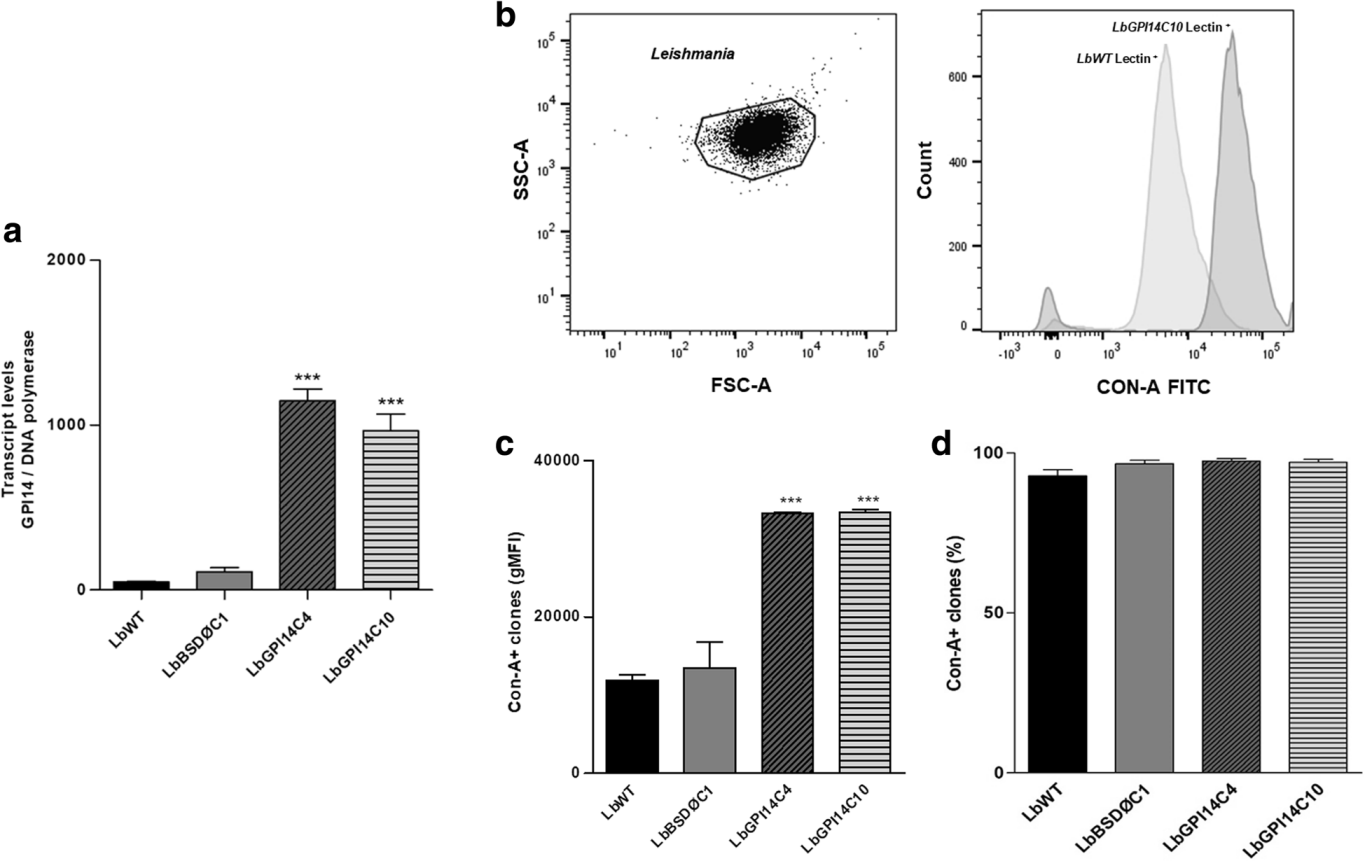
Levels of transcription of the *GPI-14* gene (**a**) and concanavalin-A (Con-A) lectin binding profile (**b**-**d**) in the wild type and GPI-14 overexpressor *L. braziliensis* lines. **a** Levels of *GPI-14* mRNA as determined quantitatively (relative to the DNA polymerase *Leishmania* gene) by real-time RT-PCR. Transcript levels ratio (*GPI-14/DNA polymerase gene*) ± standard deviations are indicated from three independent experiments. **b** Flow cytometric analysis of the differential expression of surface carbohydrate in wild-type (LbWT) and GPI14C10 clone (LbGPI14C10) of *L. braziliensis* using concanavalin-A (Con-A) lectin conjugated to fluorescein isothiocyanate (FITC). **c** The GPI-14-overexpressor clones were compared to the wild-type *L. braziliensis* line by the amount of α-D-mannosyl and α-D-glucosyl on its surface using concanavalin-A FITC agglutination profile and a significant difference is seen in the mean intensity of fluorescence (gMIF) in both clones. **d** The percentage (%) of Con-A FITC labeled parasites was used as a control parameter of efficiency. Data obtained in duplicates from at least three independent experiments were analyzed by Student’s t-test using GraphPad Prism 5.0 software. Statistically significant differences (*P* < 0.001) between wild type and GPI-14 overexpressor parasites are showed by asterisks (***). Pairwise comparisons: **a** LbWT *vs* LbGPI14C4 (*t*_(3)_ = 21.16, *P* = 0.0002); LbWT *vs* LbGPI14C10 (*t*_(4)_ = 9.130, *P* = 0.0008); **c** LbWT *vs* LbGPI14C4 (*t*_(3)_ = 25.03, *P* = 0.0001); LbWT *vs* LbGPI14C10 (*t*_(3)_ = 23.92, *P* = 0.0002)

The wild type *L. braziliensis* line was transfected with the construct pIR1BSD-GPI-14 to generate parasites overexpressing the enzyme GPI-14. This construct integrates into the *18S* ribosomal DNA small subunit locus, by homologous recombination [19]. After two weeks, the genomic DNA from the transfected clonal lines was subjected to PCR analysis with primers specific for BSD marker that confers resistance to blasticidin. The results indicated the presence of a 399 bp fragment in all blasticidin-resistant clones (data not shown), confirming successful transfection. It is important to highlight that GPI-14-overexpressing clones showed similar growth curves in comparison with the wild-type *L. braziliensis* (data not shown). These clones were subjected to quantitative real-time PCR in order to confirm overexpression of the GPI-14 enzyme. The results showed that *GPI-14* transcription levels were 23.5- and 19.8-fold higher in the transfected clones C4 and C10, respectively, derived from the wild-type *L. braziliensis* lines than in the non-transfected or empty vector transfected (LbBSDØC1) ones (Fig. 1a).

We also investigated the expression profile of the surface carbohydrates in GPI-14 transfected clones by percentage and geometric mean fluorescence intensity (gMFI) through flow cytometry of the parasites incubated with concanavalin-A (Con-A) (Fig. 1b). The results showed that clones transfected with GPI-14 express 2.8-fold more mannose and glucose residues than the non-transfected (LbWT) or empty vector (LbBSDØC1) transfected *L. braziliensis* lines, showing effective GPI-14 overexpression (Fig. 1c). A similar parasite percentage of Con-A for each *Leishmania* sample was observed, indicating the homogeneity of the parasites regarding the expression of molecules containing mannose or glucose residues (Fig. 1d). However, the mean fluorescence intensity was higher in the GPI-14 overexpressor clones, indicating that GPI-14-overexpressing clones presented higher mannose/glucose expression (Fig. 1c).

**Fig. 2:**
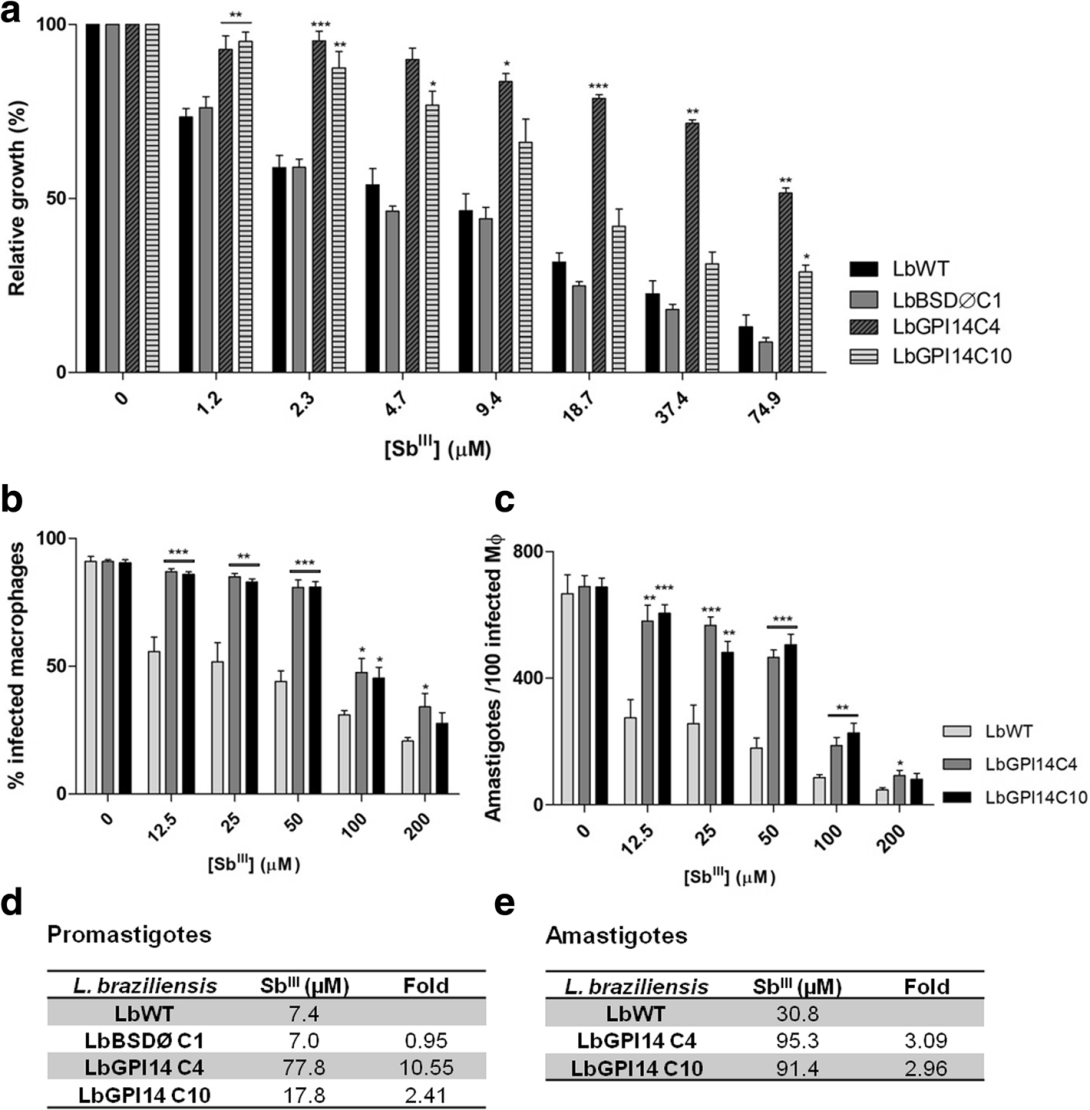
Sb^III^ susceptibility assays on the promastigotes and intracellular amastigotes of wild type and GPI-14-overexpressor *L. braziliensis* clones. **a** Promastigotes were cultured in the absence or presence of increasing Sb^III^ concentrations (1.2 to 74.9 μM) for 48 h and the percentage of relative growth was determined using a Z1 coulter counter. **b**, **c** Sb^III^ susceptibility was evaluated on amastigotes using THP-1-derived human macrophages. THP-1 macrophages infected with *L. braziliensis* lines were cultured in the absence or presence of increasing Sb^III^ concentrations (12.5 to 200 μM) for 72 h and the percentage of infected macrophages (**b**) and number of parasites per 100 macrophages (**c**) was determined. The EC_50_ (μM) values were calculated for promastigotes (**d**) and amastigotes (**e**) of LbWT, LbGPI14C4 and LbGPI14C10 and the fold change in the resistance index of overexpressor clones relative to LbWT was determined. Statistical analysis of the curves was analyzed by Student’s t-test. ‘*’ represents a statistical difference between the wild-type lines and each overexpressor clone (*P < 0.05, **P < 0.01 and ***P < 0.001; see Tables 1 and 2 for details on pairwise comparisons)

Further to this, we investigated whether overexpression of the *GPI-14* gene contributes to the antimony resistance phenotype in *Leishmania*. For this, clonal lines from *L. braziliensis* promastigotes transfected with constructs pIR1BSD (empty vector), pIR1BSD-GPI-14 and non-transfected parasites were incubated with different Sb^III^ concentrations. The EC_50_ was determined by counting the number of parasites grown in the absence or presence of this drug. The data indicated that the Sb^III^ EC_50_ values of non-transfected (LbWT) and empty vector transfected *L. braziliensis* lines were 7.4 and 7.0 μM, respectively, whereas clones 4 and 10 showed EC_50_ values of 77.8 and 17.8 μM, respectively (Fig. 2a, d) (Table 1). This result demonstrates that these clones were approximately 10.55- and 2.41-fold more resistant to Sb^III^ than the non-transfected wild-type *Leishmania* line (LbWT), respectively (Fig. 2d).

**Table 1:** Statistical analysis of Sb^III^ susceptibility assays on the promastigotes of wild type and GPI-14-overexpressor *L. braziliensis* clones

Sb^III^ (μM)	Pairwise comparisons^a^	
	LbWT *vs* LbGPI14C4	LbWT *vs* LbGPI14C10
1.2	*t*_(6)_ = 4.298, *P* = 0.0051	*t*_(4)_ = 5.548, *P* = 0.0052
2.3	*t*_(6)_ = 8.217, *P* = 0.0002	*t*_(4)_ = 4.815, *P* = 0.0086
4.7	–	*t*_(4)_ = 3.081, *P* = 0.0369
9.4	*t*_(3)_ = 5.624, *P* = 0.0111	–
18.7	*t*_(3)_ = 13.58, *P* = 0.0009	*t*_(4)_ = 1.837, *P* = 0.1400
37.4	*t*_(2)_ = 12.84, *P* = 0.0060	*t*_(2)_ = 1.699, *P* = 0.2315
74.9	*t*_(3)_ = 8.519, *P* = 0.0034	*t*_(3)_ = 3.417, *P* = 0.0419

^a^Student’s t-test

To analyze whether this antimony-resistant phenotype persists in parasite’s intracellular form, amastigotes of GPI-14-overexpressing *L. braziliensis* clones were also subjected to susceptibility assays with Sb^III^. The data showed that the numbers of infected macrophages (Fig. 2b) (Table 2) and amastigotes/100 infected macrophages (Fig. 2c) (Table 2) were higher in the GPI-14 overexpressor clones 4 and 10 than those in the non-transfected wild-type *Leishmania* line. The amastigotes from both GPI-14 overexpressor clones were about 3-fold more resistant to Sb^III^ (EC_50_ values of 91.4 and 95.3 μM for clones 10 and 4, respectively) than the wild-type line (EC_50_ 30.8 μM) (Fig. 2e). No difference in the infectivity of macrophages was observed among wild-type *L. braziliensis* line and GPI-14 overexpressor clones in the absence of Sb^III^ (Fig. 2b, c).

**Table 2:** Statistical analysis of Sb^III^ susceptibility assays on the intracellular amastigotes of wild type and GPI-14-overexpressor *L. braziliensis* clones

Sb^III^ (μM)	Percentage of infected macrophages^a^		No. of parasites per 100 macrophages^b^	
	LbWT *vs* LbGPI14C4	LbWT *vs* LbGPI14C10	LbWT *vs* LbGPI14C4	LbWT *vs* LbGPI14C10
12.5	*t*_(10)_ = 5.575, *P* = 0.0002	*t*_(10)_ = 5.364, *P* = 0.0003	*t*_(10)_ = 3.998, *P* = 0.0025	*t*_(10)_ = 5.180, *P* = 0.0004
25	*t*_(10)_ = 4.376, *P* = 0.0014	*t*_(10)_ = 4.094, *P* = 0.0022	*t*_(10)_ = 4.822, *P* = 0.0007	*t*_(10)_ = 3.284, *P* = 0.0082
50	*t*_(10)_ = 7.174, *P* < 0.0001	*t*_(10)_ = 7.865, *P* < 0.0001	*t*_(10)_ = 7.254, *P* < 0.0001	*t*_(10)_ = 7.140, *P* < 0.0001
100	*t*_(10)_ = 2.925, *P* = 0.0152	*t*_(10)_ = 3.164, *P* = 0.0101	*t*_(10)_ = 3.776, *P* = 0.0036	*t*_(10)_ = 4.558, *P* = 0.0010
200	*t*_(10)_ = 2.449, *P* = 0.0343	*t*_(10)_ = 1.524, *P* = 0.1584	*t*_(10)_ = 2.465, *P* = 0.0334	*t*_(10)_ = 1.674, *P* = 0.1250

^a^Pairwise comparisons (Student’s t-test) (Fig. 2b)

^b^Pairwise comparisons (Student’s t-test) (Fig. 2c)

## Discussion

Chemotherapy for leishmaniasis presents several problems, such as high toxicity, long treatment schedules, and the occurrence of resistant strains to pentavalent antimonials. Therefore, the need to identify drug resistance mechanisms and new molecular targets for chemotherapy of this disease is necessary.

It is well established that *Leishmania* spp. synthesizes a unique class of molecules known as phosphoglycans (PGs), including the membrane-bound lipophosphoglycan (LPG) and secreted proteophosphoglycan (PPG). PGs are essential for host-parasite interactions, such as infectivity and survival of the parasite in the human host, interaction of parasite in the host’s gastrointestinal tract, proliferation, and evasion of the vertebrate host’s immune system, amongst others [12, 20–23]. The important feature of LPG is the presence of 15 to 30 copies of a disaccharide-phosphate repeating unit Gal(β1,4)Man(α1-PO_4_), which is also found in many other molecules, such as secreted acid phosphatase, phosphoglycan and proteophosphoglycan [24]. Studies have shown that LPG repeating units are involved in the parasite’s infectious cycle [24–27]. This repeating unit named PG domain is assembled by the sequential action of the α-D-mannosylphosphate transferase (MPT) and 1,4-β-galactosyltransferase (GalT) enzymes. The MPT [24], also named mannosyltransferase (GPI-14) [28], is an essential enzyme for adding mannose on the glycosylphosphatidyl. This enzyme transfers an intact α-D-mannose-phosphate moiety from the nucleotide sugar donor GDP-Man to the glycan substrate [29]. Thus, the role of GPI-14 in the antimony-resistance mechanism was investigated in this study, given there is no previous data on this in the literature.

After stable transfection with pIR1BSD-GPI-14 plasmid, *L. braziliensis* clones showed an increased *GPI-14* mRNA expression level. In order to investigate the expression of mannose residues on the surface of these parasites, we analyzed the agglutination profile using a lectin of plant origin, concanavalin-A. Lectins, due to their interactions with specific carbohydrates, have become useful tools for elucidating cell surface differences. Studies reported the agglutination of Con-A with ligand terminals similar to α-D-mannose and α-D-glucose present on the membrane surface of *L. braziliensis* [30]. Our results showed that *L. braziliensis* clones expressed 2.8-fold more mannose and glucose residues compared to the wild-type parental line, showing effective GPI-14 overexpression.

Interestingly, an antimony susceptibility test using promastigotes showed that two clones overexpressing the GPI-14 enzyme are 2.4- and 10.5-fold more resistant to potassium antimonyl tartrate (Sb^III^) compared to the parental non-transfected line. Infection analysis using THP-1 macrophages showed that amastigotes from both GPI-14 overexpressor clones were about 3-fold more resistant to Sb^III^ than the wild-type line. These results suggest that the GPI-14 enzyme may be implicated in the Sb^III^-resistance phenotype of *L. braziliensis*. The Sb^III^-resistance mechanism in *Leishmania* is complex, multifactorial, and involves several pathways, including the entry, metabolism, efflux and/or drug sequestration [31]. The major entry route of Sb^III^ in *Leishmania* is through aquaglyceroporin 1 (AQP1), a six helices plasma transmembrane pore forming protein [32]. Downregulation, mutation and/or deletion of AQP1-encoding gene have been clearly associated with Sb resistance [8]. We hypothesize that GPI-14 overexpressor parasites presenting a larger LPG with more mannose residues could protect these parasites from Sb^III^ by sterically hindering the macromolecules’ access to the cell membrane, thus preventing entry of Sb^III^ into the cell. Consistent with these data, it has been shown that LPG presenting a high number of repeating units protects *Leishmania* from complement-mediated damage, inhibiting channel formation and lysis by the C5-9 membrane attack complex [33].

As a prerequisite for the biosynthesis of mannose(Man)-containing glycoconjugates in *Leishmania*, phosphoglycans including LPG and PPG are an ample supply of the mannose donors GDP-Man and dolicholphosphate-Man. Loss of expression of all Man-containing glycoconjugates in *L. mexicana* by targeted deletion of the genes involved in GDP-Man biosynthesis, such as phosphomannomutase and GDP-Man pyrophosphorylase, resulted in the complete loss of virulence in macrophages and mice [34, 35]. In contrast, *L. mexicana* dolicholphosphate-Man synthase gene deletion mutants presented defects in LPG, protein GPI anchor, and GIPL biosynthesis, yet retained the capacity to synthetize mannose and remained virulent in the macrophages [35]. These data indicate that mannose activation leading to GDP-Man is a virulent pathway in *Leishmania*. Our results showed that the infectivity of GPI-14 overexpressor *L. braziliensis* clones in THP-1 macrophages was similar to that of the wild-type parasites, revealing that higher mannose residues had no effect on virulence. Conversely, the presence of 2.8-fold more mannose residues in these lines overexpressing GPI-14 contributed to antimony resistance (Fig. 2b, c).

GPI-14 is a rational target for leishmaniasis chemotherapy since it is a key enzyme in the biosynthesis of LPG and GIPLs, which are important molecules in the parasite’s interaction with vertebrate and invertebrate hosts and has a structural variation in the side chain and lipid moiety compared to humans [14]. Ruhela et al. [29] described the synthesis and evaluation of new iminosugars as inhibitors of GPI-14 using microsomal membranes from *L. donovani*. Interestingly, another group developed eight derivative compounds that were docked onto GPI-14 and proposed that these antagonists would block the GPI-14 biosynthesis process [14]. These authors, through the use of modeling and molecular dynamics analysis, observed that GPI-14 of *L. major* presents multiple transmembrane regions.

## Conclusions

To our knowledge, this study is the first to show evidence that the overexpression of GPI-14 enzyme is implicated in the *L. braziliensis* Sb^III^-resistance phenotype. Since GPI-14 is a key enzyme in the biosynthesis of LPG and GIPLs, which play important roles in the parasite infectious cycle, it has significant potential as a target for new leishmaniasis treatment alternatives. Thus, the present study opens doors in the search for new membrane targets to be studied and raises questions concerning its role in the Sb^III^-resistance phenotype.

## Abbreviations

BSD: Blasticidin
GPI-14: Mannosyltransferase
Lb: *Leishmania* (*Viannia*) *braziliensis*
Sb^III^: Trivalent antimony
WT: Wild-type

## References

[1] Chappuis F, Sundar S, Hailu A, Ghalib H, Rijal S, Peeling RW (2007). Visceral leishmaniasis: what are the needs for diagnosis, treatment and control?. Nat Rev Microbiol..

[2] World Health Organization. Leishmaniasis. 2018. http://www.who.int/en/news-room/fact-sheets/detail/leishmaniasis. Accessed 10 Sep 2018.

[3] David CV, Craft N (2009). Cutaneous and mucocutaneous leishmaniasis. Dermatol Ther..

[4] Ministério da Saúde, Brazil (2017). Secretaria de Vigilância em Saúde. Departamento de Vigilância das Doenças Transmissíveis. Manual de vigilância da leishmaniose tegumentar.

[5] Alvar J, Velez ID, Bern C, Herrero M, Desjeux P, Cano J (2012). Leishmaniasis worldwide and global estimates of its incidence. PLoS One..

[6] Kumar R, Engwerda C (2014). Vaccines to prevent leishmaniasis. Clin Transl Immunology..

[7] Croft SL, Sundar S, Fairlamb AH (2006). Drug resistance in leishmaniasis. Clin Microbiol Rev..

[8] Mukherjee A, Boisvert S, Monte-Neto RL, Coelho AC, Raymond F, Mukhopadhyay R (2013). Telomeric gene deletion and intrachromosomal amplification in antimony-resistant *Leishmania*. Mol Microbiol..

[9] Mohapatra S. Drug resistance in leishmaniasis: newer developments. Trop Parasitol. 2014;4:4–9. 10.4103/2229-5070.129142 PMC3992802 24754020

[10] Frézard F, Demicheli C, Ribeiro RR (2009). Pentavalent antimonials: new perspectives for old drugs. Molecules..

[11] Berman JD, Gallalee JV, Best JM (1987). Sodium stibogluconate (Pentostam) inhibition of glucose catabolism *via* the glycolytic pathway and fatty acid beta-oxidation in *Leishmania mexicana* amastigotes. Biochem Pharmacol..

[12] Naderer T, Vince JE, McConville MJ (2004). Surface determinants of *Leishmania* parasites and their role in infectivity in the mammalian host. Curr Mol Med..

[13] Sernee MF, Ralton JE, Dinev Z, Khairallah GN, O'Hair RA, Williams SJ (2006). *Leishmania* beta-1,2-mannan is assembled on a mannose-cyclic phosphate primer. Proc Natl Acad Sci USA..

[14] Shinde S, Mol M, Jamdar V, Singh S (2014). Molecular modeling and molecular dynamics simulations of GPI14 in *Leishmania major*: insight into the catalytic site for active site directed drug design. J Theor Biol..

[15] Liarte DB, Murta SM (2010). Selection and phenotype characterization of potassium antimony tartrate-resistant populations of four New World *Leishmania* species. Parasitol Res..

[16] Moreira DS, Murta SM (2016). Involvement of nucleoside diphosphate kinase b and elongation factor 2 in *Leishmania braziliensis* antimony resistance phenotype. Parasit Vectors..

[17] Moreira DS, Monte Neto RL, Andrade JM, Santi AM, Reis PG, Frézard F (2013). Molecular characterization of the MRPA transporter and antimony uptake in four New World *Leishmania* spp. susceptible and resistant to antimony. Int J Parasitol Drugs Drug Resist..

[18] Huber W, Koella JC (1993). A comparison of three methods of estimating EC50 in studies of drug resistance of malaria parasites. Acta Trop..

[19] Robinson KA, Beverley SM (2003). Improvements in transfection efficiency and tests of RNA interference (RNAi) approaches in the protozoan parasite *Leishmania*. Mol Biochem Parasitol..

[20] Turco SJ, Descoteaux A (1992). The lipophosphoglycan of *Leishmania* parasites. Annu Rev Microbiol..

[21] Mengeling BJ, Beverley SM, Turco SJ (1997). Designing glycoconjugate biosynthesis for an insidious intent: phosphoglycan assembly in *Leishmania* parasites. Glycobiology..

[22] Späth GF, Lye LF, Segawa H, Sacks DL, Turco SJ, Beverley SM (2003). Persistence without pathology in phosphoglycan-deficient *Leishmania major*. Science..

[23] Assis RR, Ibraim IC, Nogueira PM, Soares RP, Turco SJ (2012). Glycoconjugates in New World species of *Leishmania*: polymorphisms in lipophosphoglycan and glycoinositolphospholipids and interaction with hosts. Biochim Biophys Acta..

[24] Descoteaux A, Mengeling BJ, Beverley SM, Turco SJ (1998). *Leishmania donovani* has distinct mannosylphosphoryltransferases for the initiation and elongation phases of lipophosphoglycan repeating unit biosynthesis. Mol Biochem Parasitol..

[25] Elhay M, Kelleher M, Bacic A, McConville MJ, Tolson DL, Pearson TW (1990). Lipophosphoglycan expression and virulence in ricin-resistant variants of *Leishmania major*. Mol Biochem Parasitol..

[26] McNeely TB, Turco SJ (1990). Requirement of lipophosphoglycan for intracellular survival of *Leishmania donovani* within human monocytes. J Immunol..

[27] Kelleher M, Moody SF, Mirabile P, Osborn AH, Bacic A, Handman E (1995). Lipophosphoglycan blocks attachment of *Leishmania major* amastigotes to macrophages. Infect Immun..

[28] Maeda Y, Watanabe R, Harris CL, Hong Y, Ohishi K, Kinoshita K (2001). PIG-M transfers the first mannose to glycosylphosphatidylinositol on the lumenal side of the ER. EMBO J..

[29] Ruhela D, Chatterjee P, Vishwakarma RA. 1-oxabicyclic b-lactams as new inhibitors of elongating MPT - a key enzyme responsible for assembly of cell-surface phosphoglycans of *Leishmania* parasite. Org Biomol Chem. 2005;3:1043–8. 10.1039/b418247b 15750647

[30] Dawidowicz K, Hernandez AG, Infante RB, Convit J (1975). The surface membrane of *Leishmania*. I. The effects of lectins on different stages of *Leishmania braziliensis*. J Parasitol..

[31] Jeddi F, Piarroux R, Mary C (2011). Antimony resistance in *Leishmania*, focusing on experimental research. J Trop Med..

[32] Bhattacharjee H, Rosen BP, Mukhopadhyay R (2009). Aquaglyceroporins and metalloid transport: implications in human diseases. Handb Exp Pharmacol..

[33] Puentes SM, Sacks DL, da Silva RP, Joiner KA (1988). Complement binding by two developmental stages of *Leishmania major* promastigotes varying in expression of a surface lipophosphoglycan. J Exp Med..

[34] Garami A, Ilg T (2001). Disruption of mannose activation in *Leishmania mexicana*: GDP-mannose pyrophosphorylase is required for virulence, but not for viability. EMBO J..

[35] Garami A, Mehlert A, Ilg T (2001). Glycosylation defects and virulence phenotypes of *Leishmania mexicana* phosphomannomutase and dolicholphosphate-mannose synthase gene deletion mutants. Mol Cell Biol..

